# DETERMINANTS OF RETIREMENT-AGE WOMEN’S LABOR FORCE PARTICIPATION: A RUSSIA AND THE US COMPARISON

**DOI:** 10.1093/geroni/igac059.1072

**Published:** 2022-12-20

**Authors:** Oksana Dikhtyar

**Affiliations:** Miami University, Oxford, Ohio, United States

## Abstract

Factors such as one’s health, education, marital status, and family caregiving were found to be associated with people’s retirement timing. Few studies looked at those factors separately for men and women and few cross-cultural studies were conducted on the topic. Women tend to accumulate less income and retirement savings throughout their working careers compared to men due to having more intermittent careers and lower paying jobs. Women, in general, live longer than men and are more likely to be divorced or widowed at older ages. Thus, they must spend fewer financial resources over a longer time in retirement. One possible solution is to continue employment after reaching pensionable age. Therefore, it is important to know factors that affect women’s labor force participation at or after pensionable age.This quantitative study examines the relationship between women’s personal and family factors and their labor force participation after reaching pensionable age in Russia and the U.S. Using data from the Background Questionnaire of the Program for the International Assessment of Adult Competencies (PIAAC) survey, we analyzed a sample of Russian women ages 55 and older and American women ages 66 and older.For retirement-age Russian women, having highest level of education, living in a larger household, and having spouse or partner in the labor force were positively associated with women’s labor force participation. Likewise, American women of pensionable age with more education and a working spouse were more likely to be in the labor force. Implications for policy and future research are discussed.

